# Effect of Single-Dose Imipramine on Anal Sphincter Tone in Healthy Women: A Randomized, Placebo-Controlled Study Using Anal Acoustic Reflectometry

**DOI:** 10.1007/s00192-024-05890-5

**Published:** 2024-08-21

**Authors:** Thea Christoffersen, Jonatan Kornholt, Troels Riis, David P. Sonne, Niels Klarskov

**Affiliations:** 1 Department of Clinical Pharmacology, Copenhagen University Hospital—Bispebjerg and Frederiksberg, Bispebjerg Bakke 23, 2nd Floor, 2400 Copenhagen, Denmark; 2 Department of Clinical Medicine, University of Copenhagen, Copenhagen, Denmark; 3 Department of Gynecology and Obstetrics, Copenhagen University Hospital – Herlev and Gentofte, Herlev, Denmark; 4 Present Address: Novo Nordisk A/S, Bagsvaerd, Denmark

**Keywords:** Imipramine, Randomized controlled trial, Anal canal, Anal acoustic reflectometry, Fecal incontinence

## Abstract

**Introduction and Hypothesis:**

Despite the high prevalence of fecal incontinence, existing treatment options may be inadequate. Drugs that enhance the tone of the anal sphincter complex could potentially be an effective pharmacological approach. This study investigated the effect of the tricyclic antidepressant imipramine on anal sphincter tone in healthy women, employing anal acoustic reflectometry as the evaluating method.

**Methods:**

In a double-blind, randomized, placebo-controlled crossover study, 16 healthy female volunteers were randomized to one of two treatment sequences. The participants attended two study visits separated by at least 7 days’ washout. At each visit, they received a single dose of 50 mg imipramine or matching placebo, in alternating order. We assessed the anal opening pressure under the resting state and during voluntary squeezing of the pelvic floor. Measurements were performed pre-dose and 1 h after drug administration, corresponding to the estimated time of peak plasma concentration of imipramine.

**Results:**

All participants completed the study. In total, 44% of the participants reported at least one adverse effect, primarily anticholinergic. Compared with placebo, imipramine increased anal opening pressure by 15.2 cmH_2_O (95% confidence interval [CI] 2.0–28.2 cmH_2_O, *p* = 0.03) in the resting state and 15.1 (95% CI 4.2–26.0 cmH_2_O, *p* = 0.01) cmH_2_O during squeezing.

**Conclusions:**

The findings indicate that imipramine increases anal sphincter tone in healthy women. However, further research is required to evaluate its clinical impact on individuals with fecal incontinence. This research also demonstrates the effectiveness of using anal acoustic reflectometry for assessing pharmacological effects on anal sphincter function.

## Introduction

Fecal incontinence (FI) is a prevalent, debilitating health care problem with a significant impact on the affected individuals’ quality of life [1, 2]. Despite the high prevalence of FI, treatment options remain limited [3, 4].

Off-label administration of low-dose tricyclic antidepressants (TCAs) has been explored for managing idiopathic FI [5]. However, evidence for this treatment approach is scarce. An open-label, uncontrolled study with 18 FI patients reported that a 4-week regimen of low-dose amitriptyline improved incontinence scores, reduced the frequency of rectal motor complexes, and improved the rectal-to-anal pressure ratio during these rectal motor complexes compared with baseline [6]. Although these suggested benefits of amitriptyline have not been confirmed in a double-blind, placebo-controlled setting, theoretical arguments exist for the possible effect of amitriptyline and other TCAs on anorectal continence function. TCAs may increase external anal sphincter tone by elevating serotonin and noradrenaline levels, i.e., neurotransmitters known to stimulate anal sphincter motor neuron activity [7]. Yet, assessment of anal sphincter function and evaluation of pharmacological treatments targeting sphincter dysfunction are complex challenges. The evaluation predominantly relies on conventional anorectal manometry, a method widely acknowledged to be the standard investigational approach. However, this technique is encumbered by considerable variability in its measurements. Such inconsistency hampers its utility, particularly in distinguishing between individuals with FI and those without [8].

Urethral pressure reflectometry (UPR), a measurement technique originating from urogynecology that utilizes acoustic reflectometry, offers high sensitivity and reproducibility compared with conventional catheter-based methods [9, 10]. With its demonstrated utility in the dynamic assessment of urethral sphincter function and in assessing drug efficacy for stress urinary incontinence, UPR has paved the way for the adaptation of acoustic reflectometry in anal sphincter assessment, now known as anal acoustic reflectometry (AAR) [11]. AAR has shown potential in providing reliable physiological measurements of anal sphincter function, displaying reproducibility comparable with anorectal manometry as well as demonstrating correlation with FI symptom severity [12–14].

In this paper, we evaluated the impact of imipramine on anal opening pressure (AOP) using the AAR technique, hypothesizing that imipramine would increase AOP compared with placebo. A secondary objective was to estimate outcome variability for AOP during the resting state and squeezing, facilitating sample size calculations for future trials of potential pharmacological treatments for FI.

## Materials and Methods

In this study conducted at Zelo Phase 1 Unit at Copenhagen University Hospital - Bispebjerg and Frederiksberg, Copenhagen, Denmark, we investigated the effect of a single dose of imipramine (50 mg) on urethral and anal pressure in healthy women. The findings from the urethral pressure measurements and a thorough description of the study design, detailed eligibility criteria, and methodologies have been published by Kornholt et al. [15].

The study was approved by the Regional Ethics Committee of the Capital Region of Denmark (approval number H‐17007330) and the Danish Medicines Agency (EudraCT no. 2017‐000119‐18), and was registered at www.ClinicalTrials.org (identifier NCT03102645). Further, the study adhered to the Declaration of Helsinki and Good Clinical Practice guidelines. All participants provided written informed consent upon enrolment.

### Study Design

This study was designed as a double-blind, randomized, placebo-controlled crossover study in healthy women. We recruited 16 healthy female volunteers by advertisement and randomized them to one of two treatment sequences (imipramine to placebo or placebo to imipramine). The Capital Region Pharmacy performed the balanced randomization (50% of the participants receiving imipramine at the first study visit) and prepared the numbered blinded dosing kits containing 50 mg imipramine and a visually identical placebo according to the sequence allocation list. The participants were consecutively assigned randomization numbers specifying their dosing kit. Imipramine was selected as the TCA study drug for this combined UPR and AAR study owing to its notable off-label use in managing stress urinary incontinence [16].

On each of the two study visits, separated by at least 7 days of washout, baseline measurements of the urethral and anal pressure were performed using UPR and AAR respectively. Subsequently, a single dose of 50 mg imipramine or placebo was administered to the participants. After 1 h of rest, corresponding to the mean time to peak plasma concentration for imipramine, 1-h post-dose UPR and AAR measurements were performed. The participants were interviewed about adverse events at the end of study visits 1 and 2, the start of study visit 2, and via telephone 5 days following the final study visit.

### Eligibility Criteria

Healthy women aged 18 to 55 with a body mass index (BMI) between 18.5 and 30.0 kg/m^2^, who were nonsmokers and consented not to breastfeed, become pregnant, and participate in other clinical trials during the study period, were eligible for this study. Primary exclusion criteria were pregnancy, any clinically relevant history or evidence of disease evaluated by the investigator, history of clinically significant urinary incontinence, and the use of any prescription or over-the-counter drugs in the 2 weeks before study drug administration (except for paracetamol and hormonal contraceptives) [15].

### Anal Acoustic Reflectometry Outcome Assessment

The AAR assessments and the parameters obtained from these have previously been described [17–19]. The AAR measurement catheter consists of a 45-cm polyvinyl chloride (PVC) tube with a thin, fully collapsible, polyurethane bag, 70 mm long, mounted on the distal end, which can be inflated to a maximum diameter of 5 mm. At its proximal end, the PVC tube is connected to a probe with a loudspeaker, a microphone, and another PVC tube to which a handheld syringe, used to inflate and deflate the bag, is connected. Sound waves initiated by a digital signal processor are amplified by the loudspeaker, reflected from the bag, and recorded by the microphone. By applying the Ware–Aki algorithm to the reflected sound waves, computer software (Oticon A/S Rhinometrics 5.6.1.1, Copenhagen, Denmark) calculates the cross-sectional area of every millimeter along the 70-mm-long bag and, in this way, corresponding pressure measurements and cross-sectional areas are obtained. In the further analysis, only the measurements from the point of minimal measurable cross-sectional area, representing the high-pressure zone of the anal canal, are used.

With the participant in the lithotomy position, the polyurethane bag is placed in the anal canal using a baby feeding tube as a guidewire. AAR measurements are recorded during rest and during voluntary contractions of the pelvic floor (squeeze). During the resting condition, the pressure is increased from 0 to 200 cmH_2_O over 7 s and decreased again to 0 cmH_2_O over 7 s. This cycle (inflation/deflation) is then repeated ten times, and the mean value of these ten measurements is used for analysis. Subsequently, the participant is asked to contract the pelvic floor (“as trying not to pass gas”). When familiar with the correct squeezing technique, the pressure is increased from 0 to 200 cmH_2_O in 7 s, and while the participant relaxes the pelvic floor, the pressure is decreased again. This squeezing procedure is repeated five times, and the mean value of five measurements is used for analysis. The total time to obtain a complete set of AAR measurements is approximately 4 min.

All UPR and AAR examinations were performed by a single operator (TR), whereas the AOP was calculated and agreed upon by two of the authors (TR and NK). All involved were blinded to the participants’ sequence allocation until statistical analyses were completed, implicating analysis of the treatment effect as A versus B before we requested the complete unblinding of the study from the hospital pharmacy.

### Sample Size Calculations and Statistical Analysis

Based on a prior AAR study, we anticipated a within-subject standard deviation (SD) of 21 cmH_2_O for the squeezing opening pressure [11]. With a sample size of 16 subjects, our study had a power of 80% to detect a 15 cmH_2_O difference in squeezing opening pressure between imipramine and placebo using a two-sided significance level of 0.05.

The baseline demographics of the subjects and the within-subject standard deviation (SD) were summarized descriptively. Changes in mean AOP (imipramine versus placebo) from baseline to the 1-h outcome were analyzed using *cros* analyses, which adjust for period effects. As generally recommended, we ensured a long washout between the study visits to minimize the risk of carry-over effects and omitted formal tests for carry-over [20, 21]. To investigate potential placebo effects, changes in mean AOP from baseline to the 1-h outcome on placebo days were analyzed using paired *t* tests. Safety data were summarized descriptively.

The statistical analyses for this paper were performed using RStudio version 1.0.136 (RStudio, Boston, MA, USA). Graphical plots were generated using GraphPad Prism version 9.4.1 for Windows (GraphPad Software, San Diego, CA, USA).

## Results

A total of 16 women were screened and enrolled in the study, and all of them successfully completed the study protocol. The median age among the participants was 25.5 (range 20–55) years, and median body mass index was 22.8 (range 20.4–27.6) kg/m^2^.

**Fig. 1:**
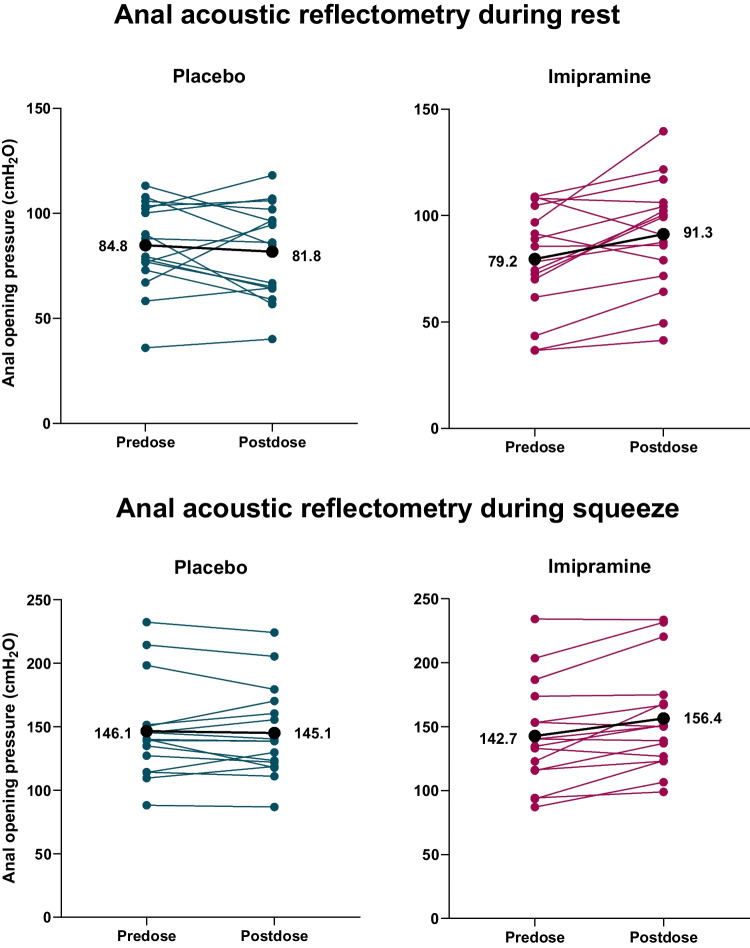
Anal opening pressure (AOP) measured using anal acoustic reflectometry. Data represent the individual mean of ten measurements per participant during rest and five measurements during squeezing. The *black line* displays the mean AOP across all participants (*n* = 16)

Mean AOP (mean of the ten measurements at rest and the five measurements during squeezing) per participant, pre-dose and in response to placebo and imipramine, are shown in Fig. 1. Compared with placebo, imipramine increased the anal opening pressure from baseline to 1-h post-dose with 15.2 cmH_2_O (95% confidence interval [CI] 2.0–28.2 cmH_2_O, *p* = 0.03) in the resting state and 15.1 cmH_2_O (95% CI 4.2–26.0 cmH_2_O, *p* = 0.01) during squeezing. Table 1 displays the estimated within-subject standard deviations.

**Table 1:** Within-subject variability

	Within-subject, within-day SD, placebo day	Within-subject, between-days SD, pre-dose imipramine and placebo days
Resting anal opening pressure, cmH_2_O	16.4	16.4
Squeezing anal opening pressure, cmH_2_O	11.8	17.5

*SD* standard deviation

The mean change (SD) from baseline to 1-h post-dose on the placebo days was −3.0 (16.4) cmH_2_O in the resting state and −1.4 (11.8) cmH_2_O during squeezing. These changes were not statistically significant when analyzed using paired *t* tests (*p* = 0.5 and 0.6 respectively).

During two study visits, 7 participants experienced 14 adverse events. One of these was reported on the placebo day (stomach cramps), the remaining after imipramine administration. The most frequent adverse events during imipramine treatment were drowsiness/sedation (5), dizziness (3), and dry mouth (3). There were no adverse events associated with the AAR procedure.

## Discussion

In this randomized, double-blind, placebo-controlled crossover study involving 16 healthy women, a single dose of 50 mg imipramine increased AOP statistically significantly compared with placebo. During the resting state of the pelvic floor and during voluntary squeezes, the estimated effect of imipramine versus placebo was 15 cm H_2_O. These observed pressure increases suggest that imipramine induces a moderate rise in the tone of the anal sphincter complex. To our knowledge, this is the first study to investigate the effect of imipramine on the anal sphincter function, and thus, the mode and site of action have not been established. However, it is likely that the rise in pressure can be attributed to increased stimulation of the striated external anal sphincter via central activation of serotonergic and noradrenergic motor neurons in Onuf’s nucleus in the sacral spinal cord [7, 22].

Physiologically, the AOP reflects the anal canal’s capacity to withstand increased pressure, serving as a metric for the closure function that contributes to continence. Previous studies assessing anal sphincter function using AAR have demonstrated that mean AOP was higher in fecally continent women compared with age-matched incontinent women [12]. Accordingly, the increased resting and squeezing AOP induced by imipramine may potentially improve continence in individuals with FI owing to weak sphincter function. However, the clinical benefit of this potential increase in sphincter tonus remains unknown, particularly when considering the possible adverse effects of imipramine. In our study, 44% of the subjects experienced at least one adverse effect following a single dose of imipramine. The majority of these were anticholinergic and central nervous system effects, resembling the side effects profile reported in longer-term studies [23]. In a broader population, particularly among the elderly and individuals with comorbidities, caution is recommended in the use of TCAs owing to their strong anticholinergic effects, potential for sedation, and risk of orthostatic hypotension [24]. The tolerability is typically better in younger users of imipramine; however, bothersome side effects such as dry mouth, increased heart rate, weight gain, and sexual dysfunction may endure during long-term treatment [23, 25, 26].

In their small, open-label study, Santoro et al. found that low-dose amitriptyline administered over 4 weeks improved FI symptoms and rectal motor activity [6]. The authors attributed these improvements to the anticholinergic properties of amitriptyline, which may prolong gut transit time, thereby enhancing water absorption and normalizing stool consistency. Such prolongation of gut transit has been observed with imipramine in individuals with irritable bowel syndrome and in healthy controls [27, 28]. A recent, randomized, double-blind, placebo-controlled study also confirmed the benefits of low-dose amitriptyline in treating irritable bowel syndrome in the primary care setting [29]. However, amitriptyline, a TCA like imipramine, may influence other pharmacological pathways as well. It may enhance anal sphincter tone by activating serotonergic and noradrenergic receptors in Onuf’s nucleus, as suggested for imipramine. Supporting this, Santoro et al. [6] reported an increase in maximum anal squeeze pressure from a pre-treatment median level of 108 (range 74–278) cmH_2_O to a median level of 123 cmH_2_O post-treatment (range not provided). Although not statistically significant, this increase in squeeze pressure matches the increase induced by imipramine in the current study. The lack of statistical significance in the study by Santoro et al. [6] can be attributed to several factors. First, its open-label, uncontrolled design compared pre-treatment and post-treatment values within the same group of individuals with FI, which is less robust than a controlled study. Additionally, the Mann–Whitney *U* tests, meant for independent samples, were used for dependent samples, reducing the statistical validity and power of the test. Furthermore, the efficacy of amitriptyline was assessed using ambulatory anorectal manometry recordings over variable timeframes, introducing considerable data variability.

In contrast, our study utilized a highly standardized AAR measurement protocol, measuring at specific time points aligned with the peak plasma concentration of imipramine, ensuring high reproducibility and low variability. With this rigorous and standardized AAR technique, we identified a statistically significant change in anal pressure, which aligns with the anticipated effects of imipramine. The within-subject (between-day) SD estimates of the AAR parameters in this study were in line with those reported in previous research (SD for resting AOP: 16.4 versus 14.3 cm H_2_O in Mitchell et al. [11] and SD for squeezing AOP: 17.5 versus 21 cm H_2_O in the same study). Additionally, mirroring previous AAR studies, the AAR assessments were feasible, averaging approximately 4 min per session, and well received by the participants. The latter is supported by the absence of adverse events associated with the AAR measurements. Moreover, our analysis confirmed that a placebo effect did not influence these outcomes. Taken together, this suggests that AAR is a reliable technique for assessing the impact of pharmacological treatments on anal sphincter function.

There are limitations to this study that merit consideration. The study population was restricted to healthy women, raising the question of whether imipramine may have a different impact on anal pressure in individuals suffering from FI. Nonetheless, previous clinical studies suggest a good correlation between the increase in urethral pressure caused by certain drugs in healthy women and their efficacy in treating women with stress urinary incontinence. Based on these findings, we believe that this correlation will likely extend to individuals with FI. Furthermore, the effects of imipramine were assessed only once post-administration. Consequently, if the peak plasma concentration of the drug was not achieved at the time of measurement, we might have overlooked its potential influence on AOP. However, earlier UPR studies successfully identified changes in urethral pressure induced by a single dose in healthy women, with the greatest increase coinciding with the estimated time to peak plasma concentration of the study drug [30].

## Conclusion

This study demonstrates that a single 50-mg dose of imipramine increases AOP in healthy women, both in the resting state and during voluntary squeezes. The observed increase in AOP suggests that imipramine might potentially improve anal sphincter function. Nevertheless, the clinical impact of these findings remains unknown.

The use of AAR in this study provided reliable and reproducible assessments of anal pressure, supporting its utility in evaluating the impact of pharmacological treatments on anal sphincter function.

## Data Availability

The data that support the findings of this study are available from the corresponding author upon reasonable request. Data are located in controlled access data storage at Copenhagen University Hospital Bispebjerg and Frederiksberg.
